# Breakthroughs in the treatment of heart failure with mildly reduced and preserved ejection fraction

**DOI:** 10.1002/clc.23846

**Published:** 2022-07-05

**Authors:** Khawaja M. Talha, Javed Butler

**Affiliations:** ^1^ Department of Medicine University of Mississippi Medical Center Jackson Mississippi USA; ^2^ Baylor Scott and White Research Institute Baylor University Medical Center Dallas Texas USA

**Keywords:** ejection fraction, heart failure, medical therapy, mildly reduced, preserved

## Abstract

Historically, only patients with a left ventricular ejection fraction (LVEF) of less than or equal to 40% were considered to have heart failure (HF). However, it was later found that patients could have elevated cardiac filling pressures and the stigmata of HF signs and symptoms with normal LVEF. This subset of patients has undergone multiple taxonomical variations and is now termed heart failure with preserved ejection fraction (HFpEF) with the lower limit of LVEF assigned as roughly ≥40%–50% in clinical trials and ≥50% in HF guidelines. Patients with LVEF 41%–49% did not clearly fit these designations but bear resemblance to both heart failure with reduced ejection fraction (HFrEF) and HFpEF. This cohort was initially assigned the term HFpEF (borderline), which has also undergone several modifications and is currently termed heart failure with mildly reduced ejection fraction (HFmrEF). Earlier landmark HF trials were heavily focused on patients with HFrEF. Only in the last 2 decades has there been an increasing focus on HFpEF with emergence of key drug therapies including sodium‐glucose cotransport‐2 inhibitors that have shown to improve outcomes across the whole LVEF spectrum. There is yet to be a focused clinical trial to determine therapeutic modalities for HFmrEF; most of the evidence has been extrapolated from subgroup analysis mostly from HFpEF trials. In this review, we provide an overview of the historical basis of HFpEF and HFmrEF and discuss key therapeutic advances in their management.

## 1 HISTORICAL BACKGROUND OF MILDLY REDUCED AND PRESERVED EJECTION FRACTION

**Figure 1:**
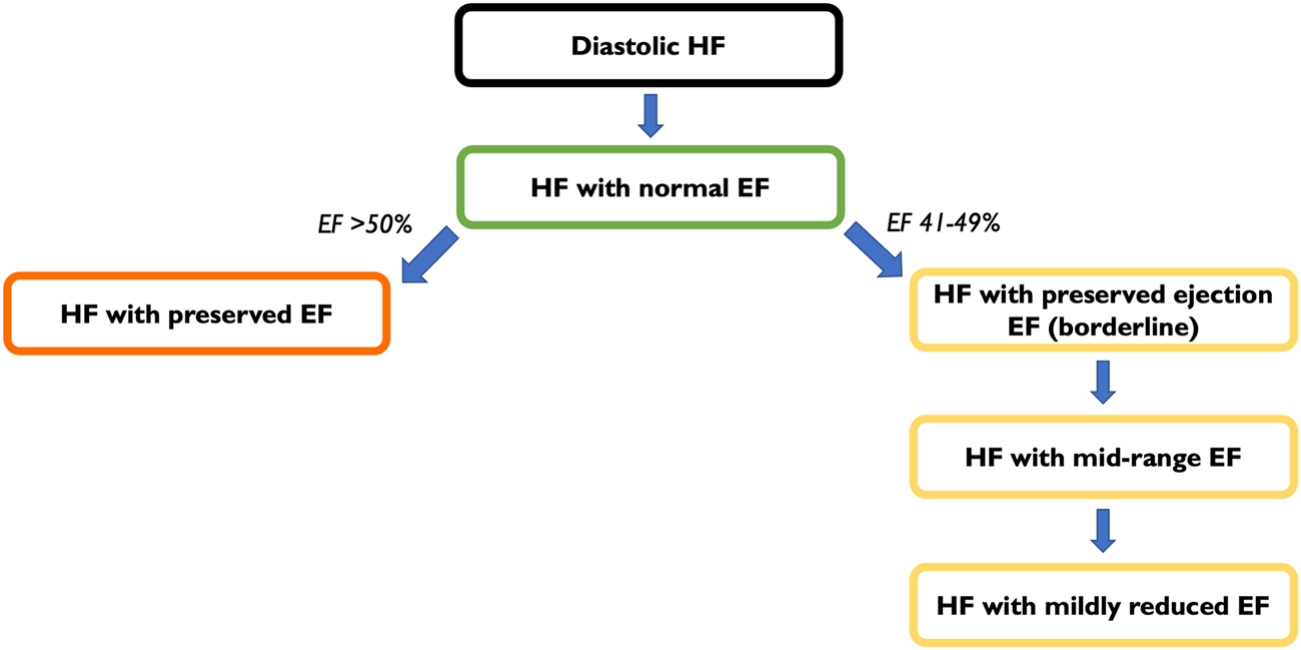
Evolution of terminologies for heart failure (HF) with ejection fraction (EF) > 40%.

The diagnosis of heart failure (HF) is currently classified based on left ventricular ejection fraction (LVEF). The LVEF cutoffs for heart failure with reduced ejection fraction (HFrEF) have not changed much since randomized controlled trials first started using LVEF as a parameter for HF diagnosis and trial enrollment, the first of which was the Veterans Administration Cooperative Study (V‐HeFT‐I) in 1986.^1^ The LVEF cutoff for HFrEF has consistently remained at <35%–45% in major clinical trials^2^, ^3^, ^4^, ^5^, ^6^, ^7^, ^8^, ^9^, ^10^ and current guidelines define HFrEF as patients with LVEF ≤40%.^11^ The term diastolic HF was initially allocated for all patients that had high intracardiac filling pressures with minimal change in the size of the left ventricular chamber who did not have a reduced ejection fraction (EF), that is, LVEF >40%.^12^ The term underwent multiple modifications over the years; it was first changed to HF with normal EF^13^ and this change in nomenclature was borne out of necessity. First, there was no reliable way to assess diastolic dysfunction using noninvasive modalities to ascertain if patients with normal EF truly had diastolic dysfunction.^14^ Second, patients with diastolic dysfunction commonly have some degree of systolic dysfunction, so labeling patients with LVEF >40% as having only diastolic dysfunction would not be entirely accurate. The candesartan in heart failure assessment of reduction in mortality and morbidity (CHARM) preserved trial^15^ was one of the first dedicated studies in patients with LVEF >40% to evaluate efficacy of an angiotensin receptor blocker (ARB). They assigned the term “preserved” ejection fraction for this cohort and following the popularity of this study, the acronym HFpEF (heart failure with preserved ejection fraction) was adopted for LVEF ≥50% universally by HF guidelines^11^, ^16^ (Figure 1).

Clinical trials for HFpEF have used varying cutoffs for enrollment; most have denoted a cutoff of >40%–50%,^15^, ^17^, ^18^, ^19^, ^20^, ^21^, ^22^ an arbitrary range that does not have a clinical or pathophysiological basis. According to current recommendations from the American Society of Echocardiography and the European Association of Cardiovascular Imaging, an LVEF of 52% in men and an LVEF of 54% in women is considered to be the lower end of normal.^23^ Hence, it would be appropriate to assume that patients with LVEF 41%–49% have significant systolic dysfunction and can potentially be categorized as HFrEF. However, this specific LVEF group has been systematically excluded from most HFrEF clinical trials as part of an enrichment strategy since this cohort has low event rates (e.g., death) requiring a higher sample size to conduct adequately powered trials.^24^ A posthoc analysis from the CHARM preserved trial^15^ revealed that ARB therapy was most effective in the LVEF 41%–49% range with diminishing efficacy with increasing LVEF. Following these findings, there was a growing interest in this LVEF range to further characterize its phenotype. It was initially designated the term HFpEF (borderline), which was formally changed to HF with mid‐range ejection fraction by the European Society of Cardiology (ESC) in 2013.^25^ Subsequent studies highlighted that HF with LVEF (41%–49%) bears more similarities to HFrEF compared to HFpEF. Evidence from the CHARM program^2^ and the European Society of Cardiology Heart Failure Long‐Term Registry (ESC‐HF‐LT) registry^26^ indicated that patients with LVEF 41%–49% had similar characteristics, including age and sex distribution, blood pressure, and ischemic heart disease to the HFrEF group. Thereafter, the term HF with mid‐range ejection fraction was changed to HF with mildly reduced ejection fraction (HFmrEF)^16^ (Figure 1).

## 2 EPIDEMIOLOGY OF MILDLY REDUCED EJECTION FRACTION

The prevalence of different HF phenotypes follows a bimodal distribution in the general population. The majority of patients fall in the HFrEF and HFpEF subgroups with a relatively smaller percentage in the HFmrEF subgroup.^27^ The CHARM program^2^ enrolled all patients with HF, irrespective of LVEF, and demonstrated an unusual unimodal LVEF distribution with ~17% patients in the LVEF 43%–52% range. The prevalence of HFmrEF is also highly variable – it accounts for 10%–25% of all patients with HF, based on registry data from multiple countries.^27^, ^28^, ^29^, ^30^, ^31^, ^32^ However, it is difficult to ascertain the true prevalence of HFmrEF as this LVEF range represents an amalgamation of patients with either HFrEF with a higher end of LVEF, HFrEF with improved LVEF, and HFpEF with LVEF at a lower end of normal or transition from HFpEF to HFrEF. It also includes patients that have been erroneously excluded from HFrEF or HFpEF categories due to inherent interobserver variability of echocardiography interpretation which can lead to variation in LVEF measurement by ±7%.^24^ A comparison of LVEF estimates in major cardiovascular trials from study sites compared to readings performed at core laboratories revealed a discrepancy in LVEF estimates of approximately 15%.^24^ Furthermore, hemodynamic factors including the patient's intravascular volume status, blood pressure, and heart rate influence the measurement of LVEF on echocardiography. Hence, LVEF of 41%–49% can be considered a snapshot across the patient's individual dynamic journey through their diagnosis of HF, instead of being regarded as a discrete entity of its own.

## 3 PHENOTYPIC AND CLINICAL CHARACTERISTICS IN MILDLY REDUCED EJECTION FRACTION

**Figure 2:**
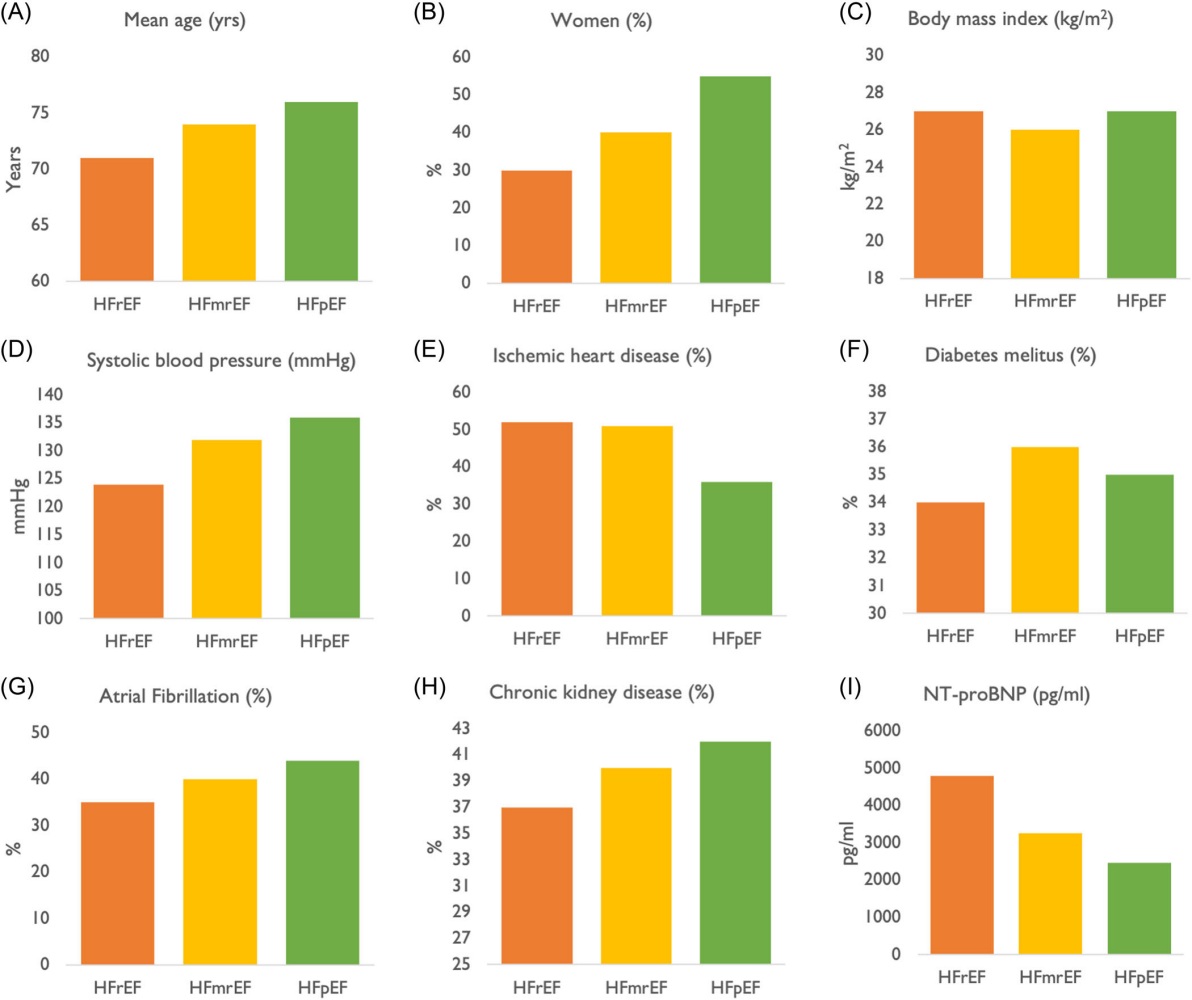
A comparison of clinical characteristics and phenotypes between HFrEF, HFmrEF, and HFpEF. Unweighted averages were obtained for all variables listed in the figure from various heart failure registries. (A) Mean age in years, data obtained from the European Society of Cardiology Heart Failure Long‐Term Registry (ESC‐HF‐LT), Swedish Heart Failure Registry (SWEDE‐HF), Get With The Guidelines Heart Failure,^36^ Organized Program to Initiate Lifesaving Treatment in Hospitalized Patients With Heart Failure (OPTIMIZE‐HF),^37^ trial of intensified (BNP‐guided) versus standard (symptom‐guided) medical therapy in chronic heart failure (TIME‐CHF),^38^ congestive heart failure cardiopoietic regenerative therapy trial (CHART‐2)^39^, and biology study to tailored treatment in chronic heart failure (BIOSTAT‐CHF)^40^ registries. (B) Percentage prevalence of women, data obtained from ESC‐HF‐LT, SWEDE‐HF, GWTG‐HF, OPTIMIZE‐HF, TIME‐CHF, CHART‐2, and BIOSTAT‐CHF registries. (C) Mean body mass index in kg/m^2^, data obtained from ESC‐HF‐LT, SWEDE‐HF, GWTG‐HF, OPTIMIZE‐HF, TIME‐CHF, CHART‐2, and BIOSTAT‐CHF registries. (D) Mean systolic blood pressure in mmHg, data obtained from ESC‐HF‐LT, SWEDE‐HF, GWTG‐HF, OPTIMIZE‐HF, TIME‐CHF, CHART‐2, and BIOSTAT‐CHF registries. (E) Percentage prevalence of ischemic heart disease, data obtained from ESC‐HF‐LT, SWEDE‐HF, GWTG‐HF, OPTIMIZE‐HF, TIME‐CHF, CHART‐2, and BIOSTAT‐CHF registries. (F) Percentage prevalence of diabetes mellitus, data obtained from ESC‐HF‐LT, SWEDE‐HF, GWTG‐HF, OPTIMIZE‐HF, TIME‐CHF, CHART‐2, and BIOSTAT‐CHF registries. (G) Percentage prevalence of atrial fibrillation, data obtained from ESC‐HF‐LT, SWEDE‐HF, GWTG‐HF, OPTIMIZE‐HF, TIME‐CHF, CHART‐2, and BIOSTAT‐CHF registries. (H) Percentage prevalence of chronic kidney disease, data obtained from ESC‐HF‐LT, SWEDE‐HF, GWTG‐HF, TIME‐CHF, and BIOSTAT‐CHF registries. (I) Mean serum levels of *N*‐terminal proB‐type natriuretic peptide (NT‐proBNP in pg/ml). Data were obtained from SWEDE‐HF, GWTG‐HF, TIME‐CHF, and BIOSTAT‐CHF registries. HFmrEF, heart failure with mildly reduced ejection fraction; HFpEF, heart failure with preserved ejection fraction; HFrEF, heart failure with reduced ejection fraction.

HFmrEF has mixed characteristics to both HFrEF and HFpEF, although it does bear more similarities with HFrEF. In observational studies performed in the US, HFmrEF was found to have similar age distribution, body mass index, and prevalence of hypertension and atrial fibrillation to HFpEF; however, distribution of sex and prevalence of ischemic heart disease resembled that of HFrEF.^33^, ^34^ Similarly, data from the ESC‐HF‐LT registry revealed a higher prevalence of ischemic heart disease and a lower prevalence of atrial fibrillation in HFmrEF, similar to the HFrEF cohort. The Swedish Heart Failure Registry^30^ also reported similar age distribution and prevalence of diabetes mellitus, chronic kidney disease, and ischemic heart disease in HFmrEF and HFrEF. Alternatively, there is data to suggest that HFmrEF mimics HFpEF in terms of clinical outcomes. In the CHARM program, investigators observed a ~40% increase in all‐cause mortality events for every 10% reduction in LVEF below 45%, while rates of all‐cause mortality and cardiovascular death remained stable with increasing LVEF over 45%.^35^ An illustration of mean estimates of several clinical characteristics and phenotypes from multiple HF registries^36^, ^37^, ^38^, ^39^, ^40^ is provided in Figure 2.

Treatment response to neurohormonal antagonism in HFmrEF (as discussed below) also suggests a similarity in pathophysiology to HFrEF. However, the circulating levels of *N*‐terminal pro‐B type natriuretic peptide (NT‐proBNP) and serum norepinephrine, markers of neurohormonal activation and sympathetic activity, in HFmrEF were found to be similar to HFpEF with significantly higher levels observed in HFrEF.^30^ A focused analysis of HF biomarkers revealed that HFmrEF possessed a more intermediate biomarker profile between the HFrEF and HFpEF phenotypes, demonstrating changes associated with both cardiac stretch, that is primarily associated with HFrEF, and inflammation, that is primarily associated with HFpEF.^30^ Hence, data pertaining to circulating biomarkers of HF in HFmrEF has so far been equivocal.

## 4 PHARMACOLOGICAL ADVANCES IN MILDLY REDUCED EJECTION FRACTION AND PRESERVED EJECTION FRACTION

**Figure 3:**
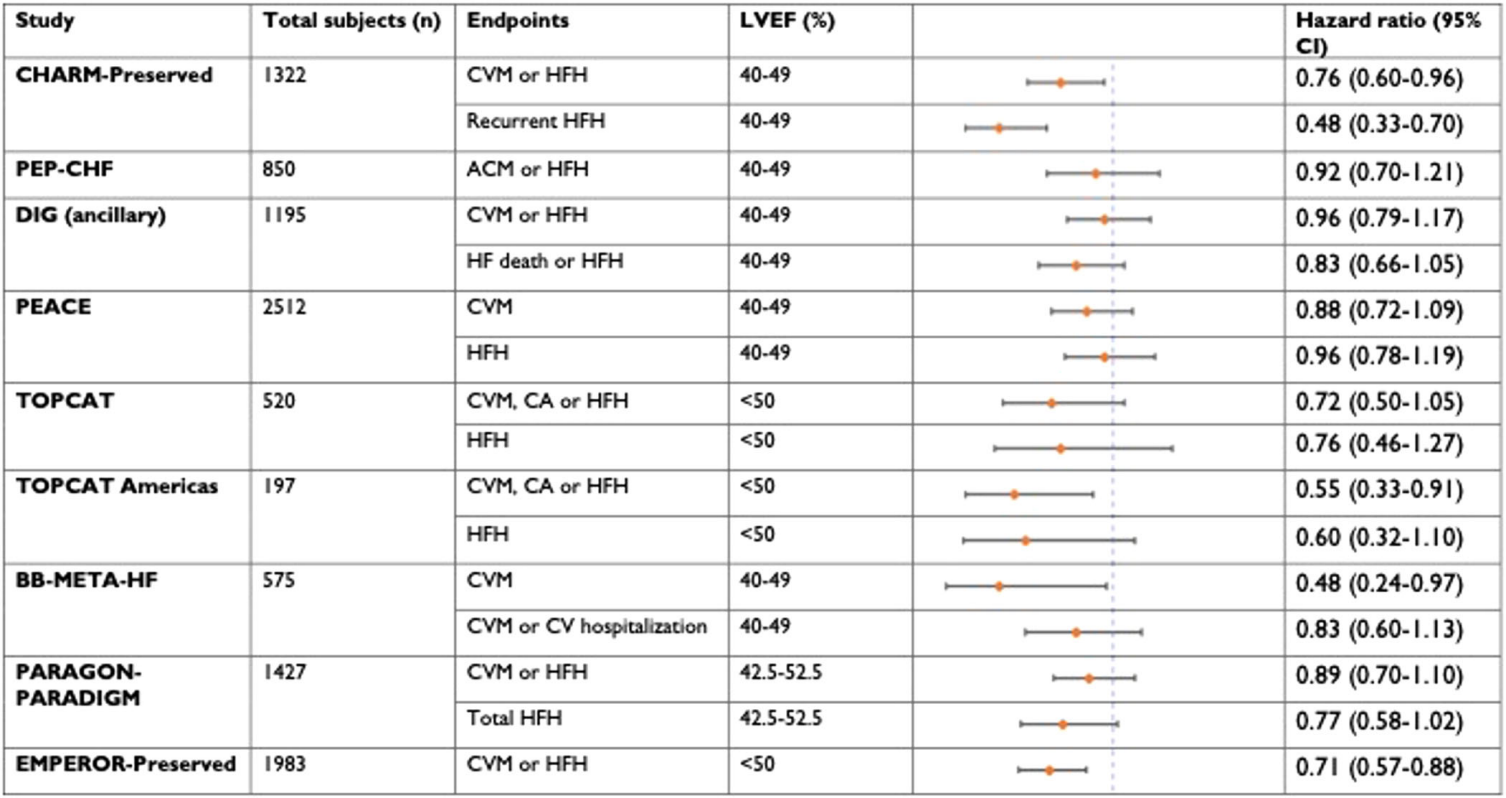
Illustration of primary endpoint results for HFmrEF from a subgroup analysis of HFpEF clinical trials. Adapted with permission from Savarese et al.^43^ ACM, all‐cause mortality; BB‐meta‐HF, beta‐blockers in Heart Failure Collaborative Group^19^, ^22^; CA, cardiac arrest; CHARM, candesartan in heart failure assessment of reduction in mortality and morbidity^15^; CI, confidence interval; CVM, cardiovascular mortality; DIG (ancillary), Digitalis Investigation Group trial^42^; EMPEROR, empagliflozin outcome trial in patients with chronic heart failure with preserved ejection fraction^10^; HF, heart failure; HFH, heart failure hospitalization; HFmrEF, heart failure with mildly reduced ejection fraction; HFpEF, heart failure with preserved ejection fraction; LVEF, left ventricular ejection fraction; PARADIGM, prospective comparison of ARNI (angiotensin receptor–neprilysin inhibitor) with ACEI (angiotensin‐converting–enzyme inhibitor) to determine impact on global mortality and morbidity in heart failure trial^6^; PARAGON, prospective comparison of ARNI with ARB (angiotensin‐receptor blockers) global outcomes in HF with preserved ejection fraction; PEACE, prevention of events with angiotensin‐converting–enzyme inhibition^41^; PEP‐HF, perindopril in elderly people with chronic heart failure^44^; TOPCAT, treatment of preserved cardiac function heart failure with an aldosterone antagonist.

HFpEF remains a considerably difficult diagnosis to manage owing to its multifaceted etiology, ranging from cardiovascular risk factors like hypertension, diabetes mellitus, and chronic kidney disease to infiltrative disorders like amyloidosis and sarcoidosis primarily causing diastolic dysfunction. Until recently, there had been a dearth of major clinical trials of pharmacological therapies for HFpEF. Management strategies focused only on targeted optimization of treatment of culprit risk factors for example, drug therapy for blood pressure control and as‐needed use of diuretics for HF exacerbation episodes to ensure optimization of patient's volume status. Although there have been several successful clinical trials for HFpEF in the past 20 years which has led to a paradigm shift in its treatment, there is no current therapy that significantly improves mortality. In contrast, there have been no dedicated clinical trials for HFmrEF thus far given its relatively recent emergence as a clinically meaningful entity. Most of the evidence for beneficial pharmacological therapy has been derived from subgroup analysis cohorts with LVEF 41%–49% from existing HFrEF and HFpEF trials^41^, ^42^ (Figure 3).

### 4.1 Angiotensin receptor blocker/angiotensin‐converting–enzyme inhibitor

The CHARM preserved trial^15^ evaluated the efficacy of Candesartan, an ARB, versus placebo in 3023 HF patients with LVEF >40%. The study found a reduction in the composite outcome of HF hospitalizations and cardiovascular mortality in the treatment arm, nearing significance based on covariate‐adjusted analysis (hazard ratio [HR], 0.86 [95% confidence interval [CI], 0.74–1.00]). This reduction was mostly driven by a significant reduction in HF hospitalizations (covariate‐adjusted HR, 0.84 [95% CI, 0.70–1.00]), with no significant reduction in cardiovascular mortality. Subgroup analysis of 1322 patients with an LVEF of 41%–49% (now considered HFmrEF) revealed that candesartan significantly reduced the risk of the composite outcome of HF hospitalization and cardiovascular death (HR, 0.76 [95% CI, 0.61–0.96]), risk of first HF hospitalization (HR, 0.72 [95% CI, 0.55–0.95]), and risk of recurrent HF hospitalizations (HR, 0.48 [95% CI, 0.33–0.70]). The Irbesartan in heart failure with preserved ejection fraction study (I‐PRESERVE)^20^ also evaluated the efficacy of an ARB versus placebo in 4128 patients with an LVEF of ≥45%. Irbesartan was not found to have a significant reduction in the composite primary endpoint of all‐cause mortality and hospitalization for a cardiovascular cause (HR, 0.95 [95% CI, 0.86‐1.05]). The perindopril in elderly people with chronic heart failure trial (PEP‐CHF)^44^ evaluated the efficacy of an angiotensin‐converting–enzyme inhibitor (ACEi) in 850 patients 70 years or older, diagnosed with diastolic left ventricular dysfunction on echocardiography with an LVEF range of 40%–50%. The study failed to demonstrate a significant reduction in the primary endpoint of all‐cause mortality and HF hospitalization (HR, 0.92 [95% Cl, 0.70–1.21]) and was attributed to a lack of sufficient primary events causing the study to achieve a power of only 35% to achieve statistical significance in the primary endpoint.

### 4.2 Beta‐blocker

A patient‐level meta‐analysis^45^ of 18 637 patients that analyzed 11 randomized controlled trials of beta‐blocker therapy in HF was performed to evaluate the drug's benefit compared to placebo in prespecified subgroups, stratified by LVEF ranges. In patients with normal sinus rhythm, there was a significant reduction in cardiovascular mortality in the LVEF 41%–49% subgroup (HR, 0.48 [95% CI, 0.24–0.97]), with a similar magnitude of treatment effect observed in subgroups with an LVEF <40%. Importantly, there was no significant reduction in cardiovascular mortality observed in the LVEF ≥50% subgroup (HR, 1.77 [95% CI, 0.61–5.14]).

### 4.3 Mineralocorticoid receptor antagonist

The treatment of preserved cardiac function with aldosterone antagonist^22^ study enrolled 3444 patients from the Americas and Russia/Georgia with LVEF >45% to assess the efficacy of spironolactone in HFpEF. The trial did not reveal a significant reduction in the composite outcome of HF hospitalizations, aborted cardiac death, and cardiovascular mortality compared to placebo (HR, 0.89 [95% CI, 0.77–1.04]). There was also no significant reduction in first HF hospitalization or all‐cause mortality. However, a posthoc analysis^46^ of outcomes stratified according to LVEF ranges in subjects enrolled in the Americas reported a significant reduction in the composite primary outcome of HF hospitalizations and cardiovascular mortality in the LVEF <50% group of 197 patients (HR, 0.55 [95% CI, 0.33–0.91]), compared to LVEF 50%–55% group of 289 patients (HR, 0.83 [95% CI, 0.56–1.25]), LVEF 55%–59% group of 422 patients (HR, 0.85 [95% CI, 0.60–1.21]) and LVEF >60% group of 858 patients (HR, 0.89 [95% CI, 0.69–1.15]). The spironolactone initiation registry randomized interventional trial in heart failure with preserved ejection fraction (SPIRIT‐HF)^18^ is an ongoing registry‐based trial evaluating the efficacy of spironolactone versus standard of care in patients with LVEF >40% for a primary composite endpoint of HF hospitalizations and cardiovascular mortality.

### 4.4 Angiotensin‐receptor blocker–neprilysin inhibitor

In the prospective comparison of angiotensin receptor–neprilysin inhibitor (ARNI) with ARB on the management of heart failure with preserved ejection fraction (PARAMOUNT)^47^ that enrolled patients with HFpEF (LVEF ≥45%), ARNI reported a significant reduction in NT‐proBNP in 12 weeks compared to valsartan. Subsequently, the prospective comparison of ARNI with ARB global outcomes in HF with preserved ejection fraction (PARAGON‐HF)^19^ trial evaluated the efficacy of the addition of neprilysin inhibition (sacubitril) to valsartan versus only valsartan therapy in 4822 patients with HFpEF defined as an LVEF of >45%. The study reported a significant reduction in the primary endpoint of HF hospitalizations and cardiovascular mortality with ARNI therapy (HR, 0.87 [95% CI, 0.75–1.01]); however, most of the benefit was observed in 2495 patients with LVEF 45%–57% (HR, 0.78 [95% CI, 0.64–0.95]), compared to 2301 patients with LVEF >57% (HR, 1.00 [95% CI, 0.81–1.23]). Women were also found to experience a significant reduction in the primary endpoint (HR, 0.73 [95% CI, 0.59–0.90]).

### 4.5 Sodium‐glucose cotransporter 2 inhibitor

More recently, the empagliflozin outcome trial in patients with chronic heart failure with preserved ejection fraction (EMPEROR‐Preserved)^10^ evaluated the efficacy of empagliflozin, a sodium‐glucose cotransporter 2 inhibitor, in 5988 patients with HFpEF defined as an LVEF of >40% over a median follow‐up of 26 months. The study reported a significant reduction in the primary endpoint of HF hospitalizations and cardiovascular mortality in the treatment group (HR, 0.79 [95% CI, 0.69–0.90]). The majority of the 21% risk reduction in the primary endpoint was attributed to a 29% risk reduction in time to first HF hospitalization. Significant improvement in the primary endpoint was observed within 18 days of drug initiation (HR at 18 days, 0.41 [95% CI, 0.17–0.99]).^48^ The study also indicated a significant decrease in total HF hospitalizations, a decrease in the slope of estimated glomerular filtration rate, and an improvement in quality‐of‐life parameters determined by Kansas City Cardiomyopathy Questionnaire (KCCQ) clinically summary scores at 52 weeks. Of note, the reduction was significant in 1983 patients with LVEF <50% (HR, 0.71 [95% CI, 0.57–0.88] and 2058 patients with LVEF 50%–59% (HR, 0.80 [95% CI, 0.64–0.99]). However, empagliflozin did not confer a significant reduction in primary endpoint in 1947 patients with LVEF >60% (HR, 0.87 [95% CI, 0.69–1.10]). A study to test the effect of empagliflozin in patients who are in hospital for acute heart failure trial (EMPULSE)^49^ revealed promising clinical benefits of initiating empagliflozin in 530 patients hospitalized with acute HF. The trial included subjects irrespective of baseline LVEF. There was a significant improvement in the primary hierarchal composite of all‐cause mortality, total HF events or time to first HF event, and KCCQ scores (win ratio, 1.36 [95% CI, 1.09–1.68]). The benefit was similar in the LVEF ≤40% group (win ratio, 1.35 [95% CI 1.04–1.75]) compared to the LVEF >40% group (win ratio, 1.39 [95% CI, 0.95–2.03.]) The dapagliflozin evaluation to improve the LIVEs of patients with preserved ejection fraction heart failure trial (DELIVER)^50^ is an ongoing multicenter randomized controlled trial evaluating the efficacy of dapagliflozin in HF patients with LVEF >40% in attenuating primary composite outcome of time to first cardiovascular event or worsening HF event with a follow‐up of ~40 months. Formal results are currently awaited but the trial has been concluded to reach its primary effectiveness endpoint.^51^

## 5 ROLE OF DEVICE THERAPIES

Data for the efficacy of device therapy in HF patients with higher LVEF is limited. Device therapies are usually aimed at supplementing cardiac contractility during systole (cardiac resynchronization therapy, left ventricular assist device, etc.) and aborting fatal arrhythmias (implantable cardioverter‐defibrillator). HFmrEF does have a component of systolic dysfunction and can potentially benefit from device therapies. A posthoc analysis from the predictors of response to cardiac resynchronization therapy trial^52^ revealed that 24% of all participants that had been enrolled in the study based on echocardiographic measurement of EF <35% actually had an EF ≥35% on repeat assessment by core laboratories. More importantly, the efficacy of cardiac resynchronization therapy in reducing left ventricular end‐systolic volumes was similar in patients on either side of the LVEF cutoff. Cardiac contractility modulation (CCM) therapy is a novel device that has been shown to improve cardiac function, exercise tolerance, and quality of life in HFrEF by applying a nonexcitatory stimulus during the absolute refractory period in the right interventricular septum in patients with HFrEF.^53^ Exploratory analysis from randomized controlled trials has concluded that the efficacy of CCM therapy is higher in LVEF 35%–45% compared to patients with LVEF 25%–34% indicating its benefit in HFmrEF.^54^ Experimental studies have found increased phosphorylation and subsequent activation of titin, a major protein in myocytes responsible for diastolic recoil, on serial endomyocardial biopsies within 3 months of CCM therapy in HFpEF.^55^ A focused trial is yet to be performed in HFpEF and HFmrEF to examine the efficacy of CCM therapy in improving clinical outcomes.

## 6 CURRENT GUIDELINE RECOMMENDATIONS

**Figure 4:**
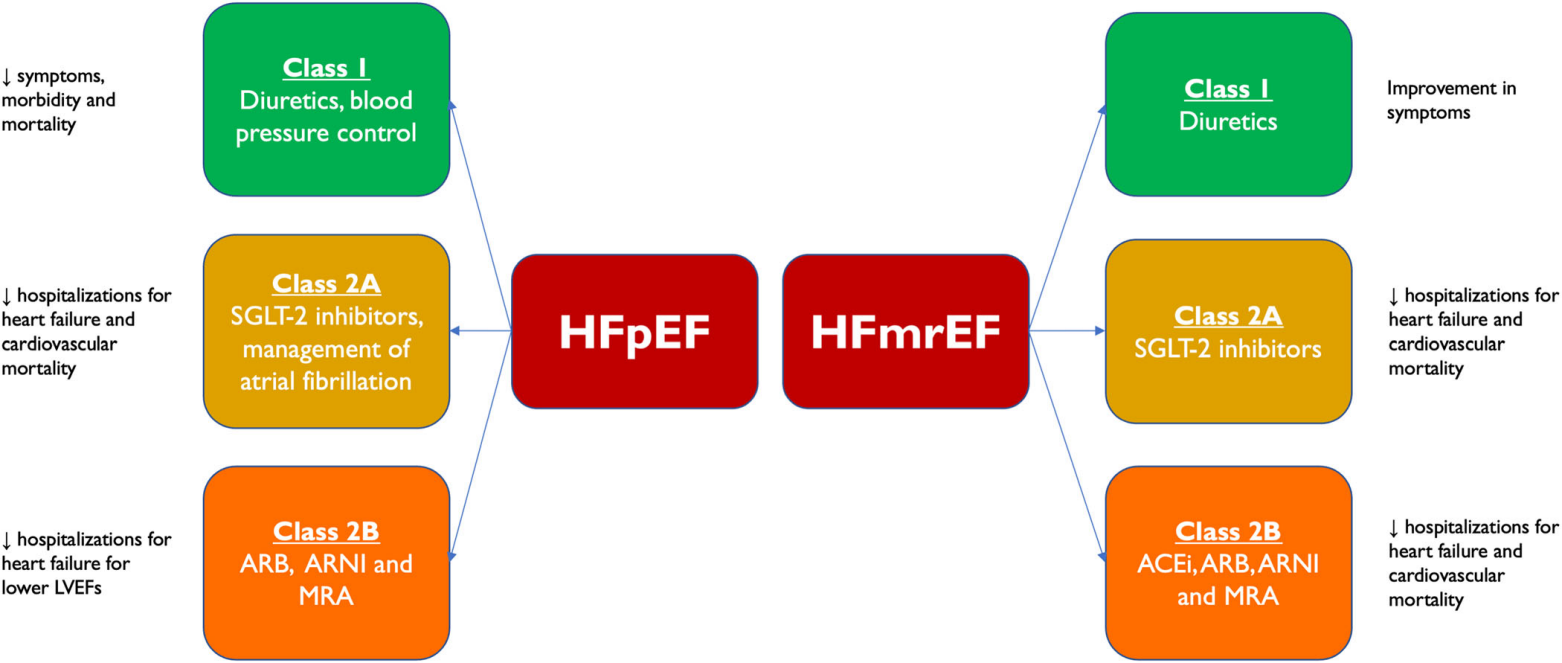
Summary of 2021 ESC and 2022 AHA/ACC/HFSA guidelines for the management of chronic HFpEF and HFmrEF. ACEi, angiotensin‐converting–enzyme inhibitor; AHA/ACC/HFSA, American Heart Association/American College of Cardiology/Heart Failure Society of America; ARB, angiotensin receptor blocker; ARNI, angiotensin receptor blocker–neprilysin inhibitor; ESC, European Society of Cardiology; HFmrEF, heart failure with mildly reduced ejection fraction; HFpEF, heart failure with preserved ejection fraction; MRA, mineralocorticoid antagonist; SGLT‐2, sodium‐glucose cotransport 2.

Figure 4 summarizes recommended therapies for HFmrEF and HFpEF by the 2021 ESC guidelines for acute and chronic heart failure^16^ and the 2022 American Heart Association/American College of Cardiology/Heart Failure Society of America (AHA/ACC/HFSA) guidelines for the management of heart failure 2022.^11^ SGLT‐2 inhibitors were not included in the ESC 2021 publication since findings from EMPEROR‐Preserved had not yet been published. The AHA/ACC/HFSA 2022 assigned a Class 2A recommendation for the use of SGLT‐2 inhibitors in both HFmrEF and HFpEF. Both ESC and AHA/ACC/HFSA denote a Class 2B recommendation for use of an ACEi, ARB, ARNI, MRA, and beta‐blocker for a reduction in HF hospitalization and death in HFmrEF. Class 2B recommendations for use of MRA, ARB, and ARNI in HFpEF are specifically indicated for patients with LVEF on the lower end of the spectrum.

## 7 CONCLUSIONS

HFpEF has garnered greater recognition over the past 2 decades and has prompted several dedicated randomized controlled trials. HFmrEF is still of growing interest as it exhibits overlapping features of both HFrEF and HFpEF of varying degrees in different populations. Although there have been multiple drug therapies that have shown to ameliorate adverse clinical outcomes in HFpEF, most of the observed benefit seems to be concentrated in patients with LVEF at the lower end of the spectrum of preserved EF and in patients with mildly reduced EF. There is growing evidence of the efficacy of neurohormonal antagonism in HFmrEF that may suggest that it is an extension of the HFrEF spectrum which has long been excluded from HFrEF trials. It would be of interest to speculate whether HFmrEF will be treated as a separate entity with its own dedicated trials or finally be merged with LVEF ≤40% group in HFrEF in future trials.

## CONFLICT OF INTEREST

Javed Butler is a consultant to Abbott, Adrenomed, Amgen, AstraZeneca, Bayer, Boehringer Ingelheim, Bristol‐Myers Squib, CVRx, G3 Pharmaceutical, Innolife, Janssen, LivaNova, Luitpold, Medtronic, Merck, Novartis, Novo Nordisk, Relypsa, Roche, and Vifor. Khawaja M. Talha declares no conflict of interest.

## Data Availability

All data generated or analysed during this study are included in this published article.
